# A Human Anti-M2 Antibody Mediates Antibody-Dependent Cell-Mediated Cytotoxicity (ADCC) and Cytokine Secretion by Resting and Cytokine-Preactivated Natural Killer (NK) Cells

**DOI:** 10.1371/journal.pone.0124677

**Published:** 2015-04-27

**Authors:** Venkateswara R. Simhadri, Milena Dimitrova, John L. Mariano, Olatz Zenarruzabeitia, Weimin Zhong, Tatsuhiko Ozawa, Atsushi Muraguchi, Hiroyuki Kishi, Maryna C. Eichelberger, Francisco Borrego

**Affiliations:** 1 Division of Biotechnology Review and Research-I, Office of Biotechnology Products Review and Research, Center for Drug Evaluation and Research, Food and Drug Administration, Silver Spring, Maryland, United States of America; 2 Immunopathology Group, BioCruces Health Research Institute, Barakaldo, Basque Country, Spain; 3 Cell Therapy and Stem Cell Group, Basque Center for Transfusion and Human Tissues, Galdakao, Basque Country, Spain; 4 Influenza Division, National Center for Immunization and Respiratory Diseases, Center for Disease Control and Prevention, Atlanta, Georgia, United States of America; 5 Department of Immunology, Graduate School of Medicine and Pharmaceutical Sciences, University of Toyama, Toyama Prefecture, Japan; 6 Division of Viral Products, Center for Biologics Evaluation and Research, Food and Drug Administration, Silver Spring, Maryland, United States of America; 7 Ikerbasque, Basque Foundation for Science, Bilbao, Basque Country, Spain; Harvard Medical School, UNITED STATES

## Abstract

The highly conserved matrix protein 2 (M2) is a good candidate for the development of a broadly protective influenza vaccine that induces long-lasting immunity. In animal models, natural killer (NK) cells have been proposed to play an important role in the protection provided by M2-based vaccines through a mechanism of antibody-dependent cell-mediated cytotoxicity (ADCC). We investigated the ability of the human anti-M2 Ab1-10 monoclonal antibody (mAb) to activate human NK cells. They mediated ADCC against M2-expressing cells in the presence of Ab1-10 mAb. Furthermore, NK cell pro-inflammatory cytokine and chemokine secretion is also enhanced when Ab1-10 mAb is present. We also generated cytokine-preactivated NK cells and showed that they still displayed increased effector functions in the presence of Ab1-10 mAb. Thus, our study has demonstrated that human resting and cytokine-preactivated NK cells may have a very important role in the protection provided by anti-M2 Abs.

## Introduction

Influenza is one of the most common viral infectious diseases in humans [1]. New strains of influenza arise due to mutations in the proteins hemagglutinin (HA) and neuraminidase (NA), a phenomenon termed antigenic drift, leading to epidemic disease [2]. More rarely, influenza strains in animals can undergo exchange of genetic material, and if infectious and transmissible in humans, viruses that result from the process of antigenic shift can cause pandemic [3]. Most licensed influenza vaccines contain HA and NA, which are highly immunogenic [4]. However, due to antigenic drift and shift, these vaccines are not designed to offer protection against newly emerging influenza variant viruses that arise through antigenic drift or shift not represented in the current vaccine. Therefore, annual reformulation of seasonal influenza vaccines is required.

Recently, the development of a vaccine based on an invariant influenza protein that would induce broad, long-lasting immunity has received much attention. Efforts to develop a “universal” influenza vaccine have focused on a number of approaches [5–7]. One target is the matrix protein 2 (M2), an ion channel of the influenza A virus, abundantly expressed at the surface of infected cells [8]. M2 is a 97 amino acid-long protein with a 23-amino acid N-terminal extracellular domain (M2e) that forms homotetramers [8,9]. Immunization with M2e or administration of anti-M2 antibodies (Abs) provided protection against challenges with influenza A virus in several animal models [10–16].

Through their role in the interplay between innate and adaptive immune responses [17], natural killer (NK) cells play a major role in eradicating virus infected cells [18,19]. Antibody dependent cell-mediated cytotoxicity (ADCC) is one of the major mechanisms wherein NK cells kill targets via the Fcγ receptor III (CD16) by recognizing and binding to the Fc portion of Abs bound to antigens on target cells [5,20,21]. In addition, ADCC-mediated NK cell activation results in the release of pro-inflammatory cytokines like interferon (IFN)-γ and tumor necrosis factor (TNF)-α, thus contributing to an effective and adequate anti-viral and Th1-response [22,23]. Furthermore, NK cells also secrete chemokines like MIP-1α, MIP-1β and RANTES, which promote the recruitment of additional NK cells and other immune cells to the site of infection [24–27]. Importantly, ADCC activity has been associated with control of the influenza infection [28]. Recent studies have also shown that NK cells have the ability to modify their behavior based on previous cytokine and/or activating receptor-mediated stimulation [29]. For example, pretreatment of NK cells with activating cytokines elicits “memory-like” properties that are defined as enhanced effector functions after re-stimulation [30,31].

In mice, vaccination with M2e generates protective Abs that mediate protection via NK cell-mediated ADCC [32]. Other studies have demonstrated that ADCC and Fc receptors play an important role in the protection provided by vaccines based on M2e and anti-M2 Abs [33–35]. Recently, it has been reported that the Ab1-10 human monoclonal Ab (mAb), that was obtained from vaccinated human volunteers, is able to recognize M2e and has the potential to induce protection against a broad range of influenza A viruses [36,37]. To delineate its effector functions, we investigated the ability of anti-M2 Ab1-10 mAb to mediate ADCC and cytokine production by human NK cells when they encounter M2-expressing cells. We show that freshly isolated NK cells exert ADCC and secrete pro-inflammatory cytokines and chemokines in the presence of Ab1-10 mAb. Furthermore, we also show that cytokine-preactivated NK cells still retain the ability of enhanced cytokine and chemokine secretion in the presence of Ab1-10 mAb. Thus, our study indicates that NK cells, in the presence of anti-M2 Abs, may play an important role in the protection provided by M2-based vaccines through ADCC, cytokine and chemokine production, and highlights the potential role of Ab1-10 mAb in identifying targets for improving influenza vaccines.

## Materials and Methods

### Purification of NK cells and cell culture

Buffy coats were collected under an institutional review board-approved protocol at the National Institutes of Health blood bank. All donors provided written informed consent. Peripheral blood mononuclear cells (PBMCs) were obtained by ficoll density centrifugation. NK cells were purified from PBMCs using a negative selection kit (Stem Cell Technologies). The purity (>95%) of NK cells was determined by flow cytometry. Purified NK cells were cultured in Iscove´s modified Dulbecco´s medium supplemented with 10% human serum, L-glutamine, sodium pyruvate and non-essential amino acids. Cytokine-preactivated NK cells were generated as previously described [30,31]. Briefly, purified NK cells (2–3 x 10^6^ cells/ml) were plated and treated for 16 hours with IL-12 (10 ng/ml), IL-15 (10 ng/ml) and IL-18 (50 ng/ml). Then, cells were extensively washed and cultured with low concentrations of IL-15 (1 ng/ml). The control cells (non-preactivated) were always in the presence of IL-15 (1 ng/ml). Every 2–3 days, half of the media was replaced with fresh medium. After 7–14 days of culture, cells were used for functional studies.

The 293FT cell line was obtained from ATCC. The generation of 293FT cells stably transfected with M2 (M2-293FT) was previously described [38]. This cell line was transfected with a construct containing the full-length M2 open reading frame from human H3N2 virus A/Panama/2007/1999. The 293FT and M2-293FT cells were cultured in Dulbecco modified Eagle medium supplemented with 10% fetal bovine serum, L-glutamine, penicillin, streptomycin, sodium pyruvate and non-essential amino acids.

### Influenza virus infection

A549 cells, a human alveolar adenocarcinoma epithelial cell line, were infected with H1N1 (A/California/07/2009) virus at 0.5 MOI (multiplicity of infection). Briefly, adherent A549 cells were thoroughly washed and incubated with H1N1 viral particles for one hour in serum-free media (Opti-MEM-Invitrogen). Then, viral particles were removed by washes and serum free media (Opti-MEM + 1X pen-strep + 1 μg/ml TPCK-Trypsin) was added to the cells and cultured for another 16 hours. After that period of time, cells were harvested using TrypLE (Invitrogen), washed and ready to use for flow cytometric and ADCC experiments.

### Flow cytometric analysis

293FT and M2-293FT cells were incubated with mouse anti-M2 14C2 mAb (Santa Cruz) and human anti-M2 Ab1-10 mAb [37] for 45 minutes on ice. After extensive washes, cells were incubated for 20–30 min on ice with specific fluorochrome conjugated secondary Abs. After several washes, cells were acquired on a BD LSRII flow cytometer (BD Biosciences) and analyzed using FlowJo software (Tree Star). A comparable procedure was followed for the detection of M2 on influenza infected A549 cells. Both 293FT and M2-293FT cells exhibited very similar side scatter and forward scatter, indicating that the expression of M2 did not significantly changed the size and complexity of 293FT cells (S1 Fig). Similarly, A549 and influenza infected A549 cells showed the same side scatter and forward scatter (S1 Fig).

### Antibody-dependent cell-mediated cytotoxicity (ADCC) assay

NK-cell mediated cytotoxicity was performed using Europium Release Assay following the manufacturer’s instructions (PerkinElmer). Briefly, europium-labeled 293FT and M2-293FT target cells were co-cultured with NK cells at different ratios in the presence or absence of Ab1-10 mAb (10 μg/ml). After 4-hour incubation, europium signals were measured in the supernatants. The percentage of cytotoxicity was determined using the formula: [experimental release—spontaneous release] / [maximal release—spontaneous release] X 100. On the other hand, influenza infected A549 cells were co-cultured with human NK cells from healthy donors at 10:1 ratio in the absence or presence of Ab1-10 mAb (2.5 μg/ml). Target cell lysis was measured using the Bioluminescence Cytotoxicity assay aCella-TOX (Cell Technology) following the manufacturer’s protocol. The percentage of specific lysis on the Y-axis was calculated with the formula: [Sample—Control Target spontaneous release—Control NK cells alone] / [Control maximum release—Control Target spontaneous release] X 100.

### Cytokine and chemokine secretion assay

NK cells were mixed with 293FT and M2-293FT cells at a 1:2 (E:T) ratio in 200 μl of medium in the presence or absence of Ab1-10 mAb (10 μg/ml). After 24-hours of incubation at 37°C, the culture supernatants were harvested and frozen at -80°C. Later, supernatants were thawed on ice and analyzed for cytokine and chemokine using the BD Biosciences Cytometric Bead Arrays following the manufacturer’s protocol. Samples were acquired on BD LSRII flow cytometer and analyzed using BD FCAP Array software.

### Statistical analysis

Data were analyzed using GraphPad Prism software. The data were plotted as bar graphs, and pair wise comparisons were examined by a paired Student’s *t*-test. NS: not significant; * P<0.05, ** P<0.01, ***P<0.001, ****P<0.0001.

## Results

### Human Ab1-10 mAb recognizes M2e and induces NK cell-mediated ADCC

In addition to neutralizing Abs (nAbs), other Abs against influenza virus can mediate other functions such as activating innate immune responses and killing influenza infected cells by several mechanisms, such as ADCC [5,28,39–41]. Previously, we isolated from a healthy donor, and then manufactured the anti-M2 Ab1-10 mAb in CHO cells [36,37]. The Ab1-10 mAb binds to cells transiently transfected with a M2 construct with an M2e consensus sequence and M2 from H5N1 and 2009 H1N1. In addition, Ab1-10 mAb is able to inhibit influenza virus replication in an *in vitro* plaque-reduction assay [36,37].

**Fig 1:**
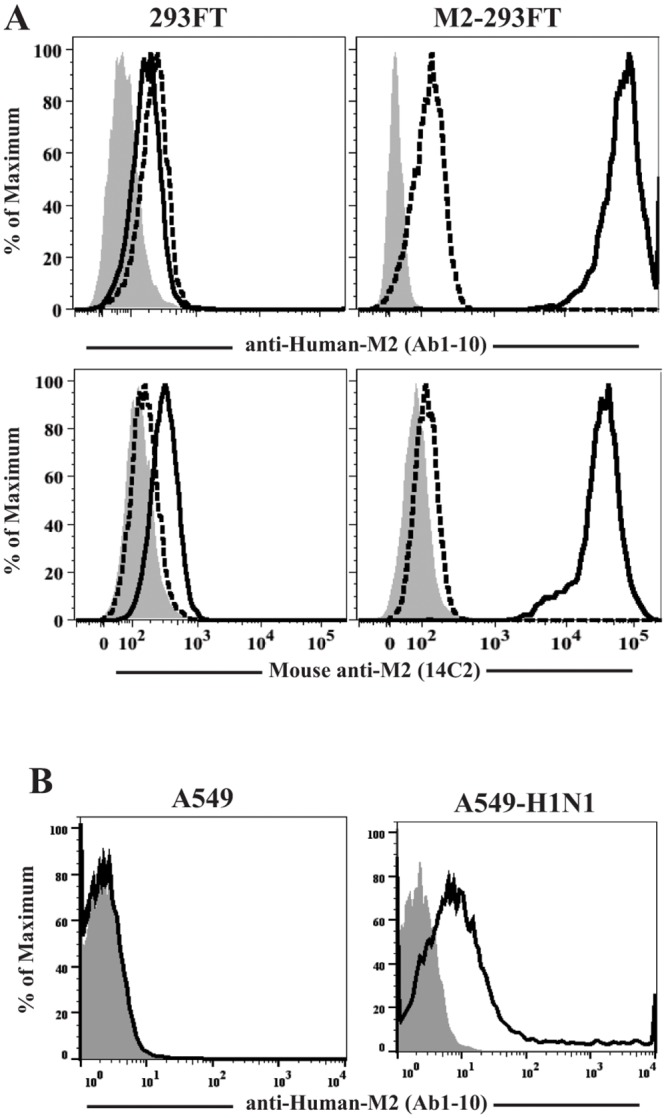
Human Ab1-10 mAb recognizes M2e on animal cells. (A) 293FT cells (left) and M2-293FT cells (right) were cell surface stained with the human anti-M2 Ab1-10 mAb (upper panel) and the mouse anti-M2 14C2 mAb (lower panel) and analyzed by flow cytometry. Shaded histograms represent unstained cells. The binding of the isotype controls (dotted line) and anti-M2e specific mAbs (black line) is shown. (B) Uninfected A549 (left) and influenza infected (right) A549-H1N1 cells were cell surface stained with the human anti-M2 Ab1-10 mAb. The binding of secondary Ab alone (grey histogram) and the binding of Ab1-10 mAb (black line histogram) is shown.

The goal of this study is to determine if this Ab1-10 mAb is able to induce NK cell-mediated ADCC. For this purpose, we made use of the M2-293FT cells, which have been successfully used as a method to quantitatively asses the specificity and cross-reactivity of anti-M2 Abs after infection or vaccination [38]. First, we tested if Ab1-10 mAb is capable of binding M2e on M2-293FT cells. Results showed that Ab1-10 mAb bound M2-293FT, but did not bind the parental 293FT cell line. As a positive control, we confirmed previous results showing that the mouse anti-M2 14C2 mAb is also able to recognize M2e on the surface of M2-293FT cells (Fig 1A). These results demonstrate that Ab1-10 mAb recognizes M2 expressing stable transfectants M2-293FT cells, and thus can be used for further functional studies. In addition, we also tested the expression of M2 in the more relevant A549 cells, a lung adenocarcinoma alveolar basal epithelial cell line. For that purpose, we infected the A549 cells, an adenocarcinoma alveolar basal epithelial cell line, with the H1N1 (A/California/07/2009) virus and demonstrated that Ab1-10 mAb is able to recognize M2 expressed on the surface of influenza infected cells (Fig 1B).

**Fig 2:**
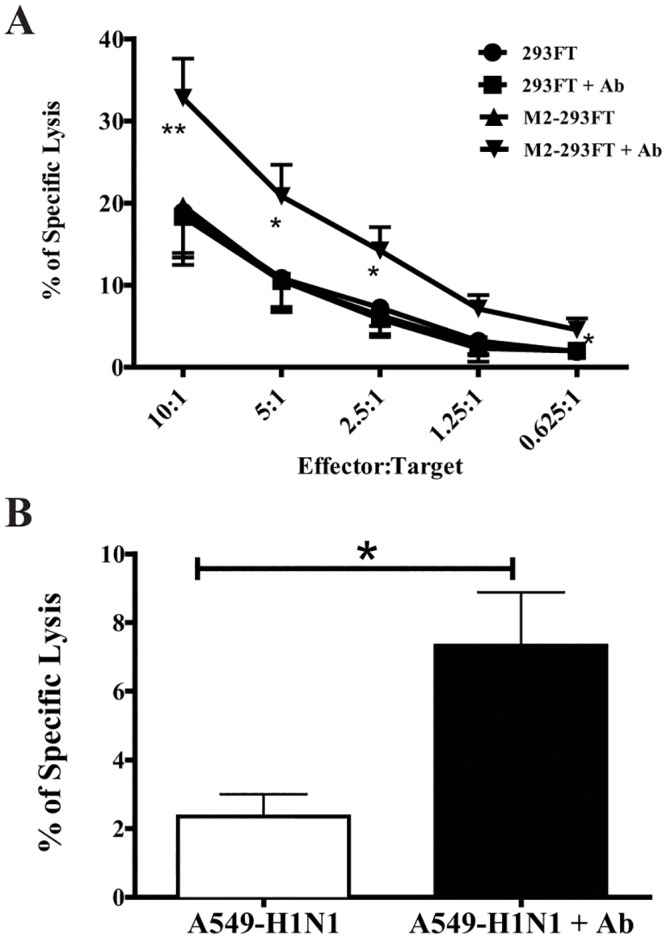
The anti-M2e Ab1-10 mAb induces freshly isolated NK cell-mediated ADCC. (A) Effectors (freshly isolated NK cells) and targets (293FT and M2-293FT cells) were co-incubated at different ratios either in the presence or absence of Ab1-10 mAb (Ab). The percentage of specific lysis is shown. Error bars represent the standard error of the mean (SEM) from five independent experiments with freshly isolated NK cells from five donors (* P<0.05, ** P<0.01). (B) Influenza infected A549 cells were co-cultured with human NK cells from healthy donors in the absence or presence of Ab1-10 mAb (Ab). The percentage of lysis is shown. Error bars represent the SEM from four independent experiments with freshly isolated NK cells from four donors (* P<0.05).

Human Abs of the IgG1 and IgG3 isotypes are capable of inducing NK cell-mediated ADCC; it is well known that licensed therapeutic Abs whose mechanism of action involves ADCC are of the IgG1 isotype [42]. Since the manufactured Ab1-10 mAb is an IgG1, we determined its ability to induce NK cell-mediated ADCC. Freshly isolated NK cells were used at different E:T ratios as effector cells in an ADCC assay against 293FT and M2-293FT target cells. NK cells are able to equally kill 293FT cells and M2-293FT cells, indicating that the expression of M2 on target cells has no effect on their susceptibility to NK cell-mediated natural cytotoxicity. However, if we added Ab1-10 mAb to the cytotoxic assay, we found that while the killing of 293FT cells is not affected, there is approximately a 65% increase in the specific lysis of M2-293FT cells, indicating that Ab1-10 mAb is inducing NK cell-mediated ADCC against M2-expressing target cells (Fig 2A). Very importantly, we also showed that Ab1-10 mAb has the potential to induce NK cell mediated ADCC against A549 influenza infected cells (Fig 2B).

### Ab1-10 mAb-induced increased cytokine and chemokine secretion by freshly isolated NK cells

**Fig 3:**
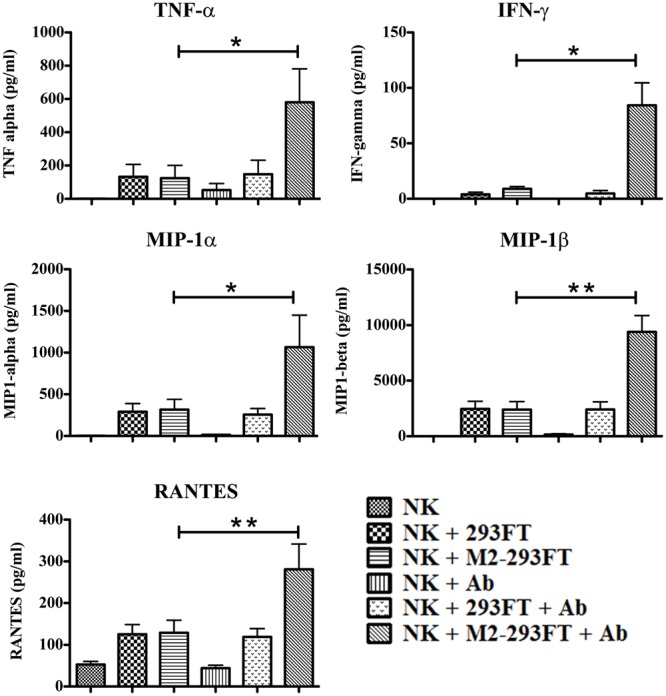
Cytokine release by freshly isolated NK cells in the presence of Ab1-10 mAb. Freshly isolated NK cells were either left untreated or co-cultured with both 293FT cells and M2-293FT cells in the presence and absence of Ab1-10 mAb (Ab). Culture supernatants were harvested and tested for the secretion of human cytokines and chemokines using flow-cytometric bead analysis. The values on the y-axis correspond to the concentrations of TNF-α, IFN-γ, MIP-1α, MIP-1β and RANTES in pg/ml. Graph bars represent the average ± SEM. Data shown are from five independent experiments with freshly isolated NK cells from five donors (* P<0.05, ** P<0.01).

In addition to killing, target recognition by NK cells also results in the secretion of chemokines and cytokines [24]. The interaction between targets and NK cells involves a multitude of ligands and receptors. Fauriat et al. studied the minimal requirements for chemokine and cytokine secretion by human NK cells. They showed that the release of chemokines occurred when CD16, 2B4, or NKG2D were engaged, whereas induction of TNF-α and IFN-γ required the engagement of two or more receptors [24]. Although we have not characterized the ligand-receptor pairs involved in the interaction of NK cells with 293FT cells, clearly there are certain ligand-receptor interactions as shown by the fact that untransfected 293FT cells are still sensitive to NK cell mediated cytotoxicity, which is significantly increased when 293FT cells express M2 and Ab1-10 mAb is incorporated into the assay (Fig 2A). In our experimental system, both CD16 and other receptors are engaged. Therefore, we sought to investigate if during ADCC with Ab1-10 mAb there is a higher production of chemokines and cytokines by NK cells. We focused on IFN-γ and TNF-α because they are very important for a successful immune response during influenza infection [22,23]. Results showed that, while there is a small production of these two cytokines when resting NK cells encounter both 293FT and M2-293FT cells, a significant increase in the production of IFN-γ (9 fold increase) and TNF-α (3.9 fold increase) occurred when NK cells encountered M2-293FT cells, but not 293FT cells, in the presence of Ab1-10 mAb (Fig 3).

Similarly, we also observed a surge in the secretion of the chemokines RANTES, MIP-1α and MIP-β, when comparing chemokine production by NK cells that encounter 293FT cells versus NK cells interacting with M2-293FT cells in the presence of Ab1-10 mAb (Fig 3). Specifically, we observed a 3.3 fold increase, a 3.9 fold increase and a 2.2 fold increase, in the secretion of MIP-1α, MIP-1β and RANTES, respectively. The secretion of these chemokines is very important for the recruitment of immune cells to the site of infection [25,27]. Altogether, our data indicate that Ab1-10 mAb is very effective in promoting cytokine and chemokine secretion by resting NK cells during the recognition of M2-expressing cells.

### Cytokine-preactivated NK cells also exhibit increased Ab1-10 mAb-mediated ADCC and cytokine secretion

**Fig 4:**
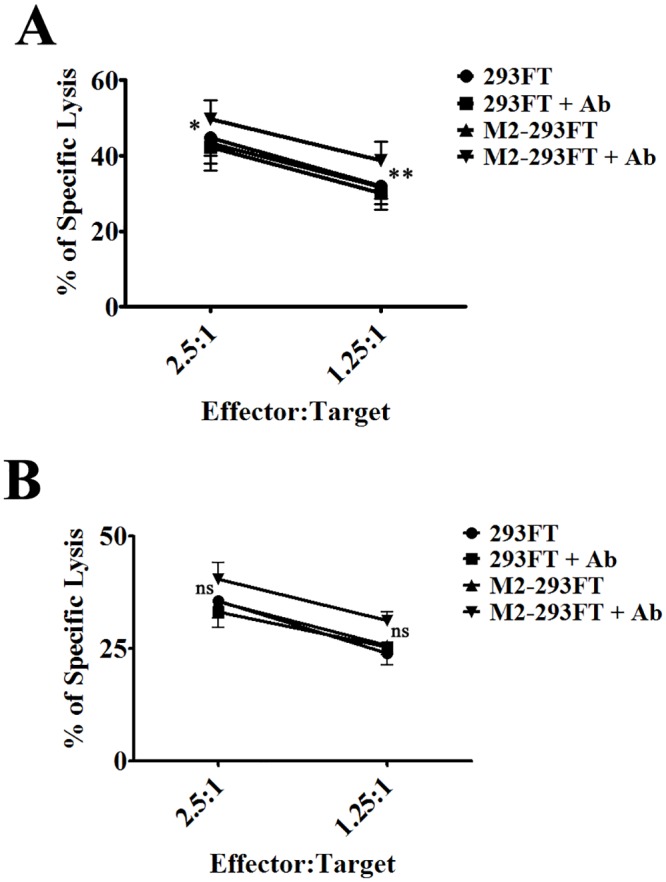
The anti-M2e Ab1-10 mAb induces cytokine-preactivated NK cell-mediated ADCC. 293FT or M2-293FT cells were used as targets and control (cultured with low concentrations of IL-15) NK cells (A) and cytokine-preactivated (pretreated with IL-12, IL-15 and IL-18) NK cells (B) were used as effector cells in an ADCC assay. Effector and target cells were co-incubated at different ratios either in the presence or absence of Ab1-10 mAb (Ab). The percentage of specific lysis is shown. Error bars represent the SEM from four independent experiments with NK cells from four donors.

**Fig 5:**
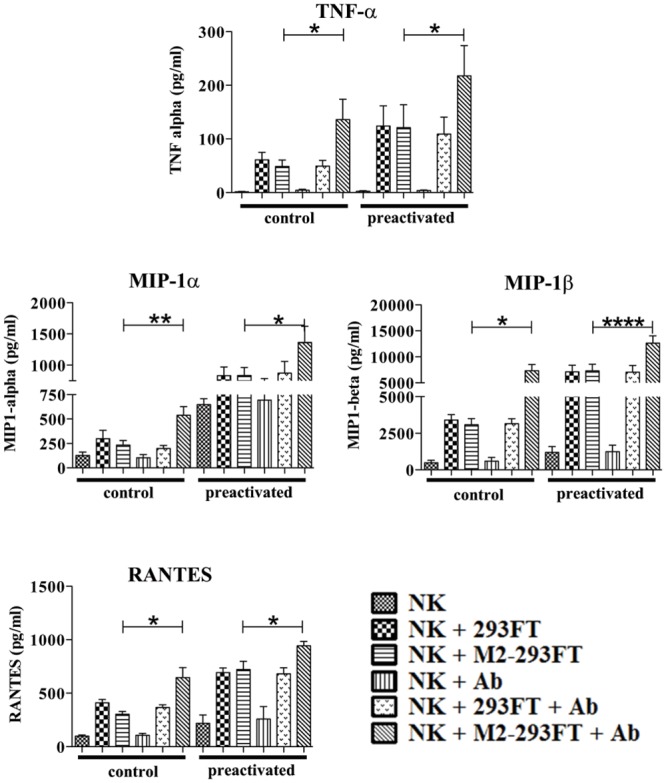
Cytokine release by cytokine-preactivated NK cells in the presence of Ab1-10 mAb. Control (cultured with low concentrations of IL-15) NK cells and cytokine-preactivated (pretreated with IL-12, IL-15 and IL-18) NK cells were co-cultured with both 293FT cells and M2-293FT cells in the presence and absence of Ab1-10 mAb (Ab). The cell culture supernatants were harvested and subjected to cytometric bead analysis for cytokine release measurements. The values on the y-axis correspond to the concentrations of TNF-α, MIP-1α, MIP-1β and RANTES in pg/ml. Graph bars represent the average ± SEM. Data shown are from independent experiments five independent experiments with NK cells from five donors (* P<0.05, ** P<0.01 ****P<0.0001).

Although NK cells have been traditionally categorized as members of the innate immune system, recent research has shown that they have the ability to change their behavior based on previous cytokine and/or activating receptor-mediated stimulation [29]. They are able to mediate recall responses to haptens and viruses [29,43,44]. Furthermore, *ex vivo* preactivation of NK cells with activating cytokines, such as IL-12, IL-15 and IL-18, elicits “memory-like” properties that are defined as enhanced effector functions after re-stimulation [30,31]. Since respiratory illnesses like influenza and respiratory syncytial virus (RSV) infections often generate a cytokine burst of pro-inflammatory cytokines that, at least hypothetically, are able to activate NK cells, we sought to determine whether cytokine-preactivated NK cells also exhibited an enhanced ADCC and cytokine production after interaction with M2-expressing cells in the presence of Ab1-10 mAb (Figs 4 and 5).

Human cytokine-preactivated NK cells were generated by overnight treating freshly isolated NK cells with a combination of IL-12, IL-15 and IL-18, and then maintained with low doses of IL-15 for 1–2 weeks. As control, we maintained untreated NK cells with low doses of IL-15 for another 1–2 weeks. As with freshly isolated NK cells, we observed that the presence of Ab1-10 mAb induced an increase in the killing of M2-293FT cells, when compared with the killing of M2-293FT cells in the absence of Ab1-10 mAb and of 293FT cells. As expected, this happened both with the cytokine-preactivated NK cells and with the control cells (Fig 4). Next, we examined the secretion of cytokines and chemokines (Fig 5). Secretion of IFN-γ by cytokine-preactivated NK cells was not increased in the presence of Ab1-10 mAb (data not shown), probably because they already reached the maximum amount of IFN-γ production. Importantly, we observed that the levels of TNF-α, MIP-1α, MIP-1β and RANTES secretion were increased when cytokine-preactivated NK cells interacted with M2-293FT cells in the presence of Ab1-10 mAb, but not with 293FT cells. In all conditions the cytokine and chemokine production by cytokine-preactivated NK cells was higher than the production by control NK cells, indicating that we effectively generated NK cells with enhanced effector functions. Thus, all together our results indicate that Ab1-10 mAb is able to increase the cytokine-preactivated NK cell-mediated effector functions.

## Discussion

After infection, NK cells are recruited to the lung and play an important role in the immune response against influenza [45]. Both through the interaction with the virus and with viral-infected cells, NK cells secrete cytokines and chemokines that are very important for the containment of the virus, the recruitment of other immune cells and tissue regeneration [45]. Direct recognition of infected cells can be achieved by the interaction of influenza proteins expressed on infected cells with NK cell activating receptors, such as NKp46 and NKp44 [46]. In addition, NK cells can recognize and eliminate infected cells that are decorated with Abs against viral proteins through an ADCC mechanism [5]. Several studies have shown that there is *in vivo* protection from influenza infection that is mediated by ADCC [28,39]. Here, we have shown that the anti-M2 Ab1-10 mAb is capable of mediating ADCC against cells transfected with the M2 protein and also against influenza infected cells. Although in this later situation freshly isolated NK cells do not efficiently kill infected A549 cells, we reproducibly found an increase in their cytotoxic activity if Ab1-10 mAb was present in the assay, which highlights the physiological relevance of our findings. Therefore, our results along with the ability of Ab1-10 mAb to suppress the spread of the virus from cell to cell through recognition of the first eight highly conserved residues of M2e [37], emphasizes the potential of including the M2 protein in the design of universal influenza vaccines.

In mice, it has been demonstrated that NK cells are able to develop specific memory against antigens from influenza [47]. In addition, during respiratory infection, mouse NK cells get activated and proliferate in the bone marrow (BM), and then migrate to the lung [48]. After becoming activated, NK cells in the airway participate in viral clearance by killing infected cells and secreting cytokines and chemokines. In addition, cytokines released in the lung may also activate both resident and newly recruited NK cells. These NK cells are long-lived, bearing a resemblance to the previously described cytokine-induced “memory-like” or preactivated NK cells [29]. Nonetheless, more studies are required to demonstrate that activated and proliferating NK cells in the BM, and those activated in the lung, are equivalent to the cytokine-induced “memory-like” NK cells. Even so, it is relevant that Ab1-10 mAb is able to mediate ADCC against M2-expressing target cells by both resting and cytokine-preactivated human NK cells, underlining the potential of this mAb for its use as a therapeutic tool during influenza infection.

In addition to Ab1-10 mAb-mediated enhancement of the direct killing of M2-expressing cells, we have also shown that there is an enhanced secretion of cytokines and chemokines by activated NK cells. TNF-α and IFN-γ promote the development of an effective Th1 response that is necessary for the successful clearance of the virus [22,23], and chemokines secreted by NK cells also have an important role in the recruitment of other immune cells to the site of infection [24–27]. For example, the Ab1-10 mAb-mediated NK-cell derived secretion of chemokines may have a very important role in recruiting CD8+ memory T cells in a CCR5 dependent manner. In this regard, reduction in memory T cells recruitment led to an impaired control of viral replication during the initial stages of a secondary response [49]. The molecular basis for the enhanced effector functions of cytokine-preactivated or “memory-like” NK cells are not well understood. An increased expression of CD16 in cytokine-preactivated NK cells could explain their ability to produce higher amounts of cytokines and chemokines when they are incubated with M2 expressing cells in the presence of Ab1-10 mAb. However, we have measured CD16 expression and found no significant differences between cytokine-preactivated and control NK cells (data not shown).

We have only characterized the role of NK cells in Ab1-10 mAb-mediated ADCC against M2-expressing cells. However, at the site of infection there are other cell types, such as macrophages and neutrophils that are also capable of mediating ADCC. For example, mouse alveolar macrophages and Fc receptors play a very important role in the protection provided by a vaccine based on M2e [33]. Therefore, the role of other cell types in Ab1-10 mAb-mediated ADCC deserves additional studies.

## Supporting Information

S1 Fig Dot plots of forward and side scatter of the 293FT cells and 293FT-M2 cells (upper panels), and uninfected and influenza infected A549 cells (lower panels). Both 293FT cells and 293FT-M2 cells exhibited very similar side scatter and forward scatter, indicating that the expression of M2 did not significantly changed the size and complexity of 293FT cells. Similarly, overnight influenza infection did not significantly change the size and complexity of A549 cells.
(DOCX)
