# Supplementary material for: A Human Anti-M2 Antibody Mediates Antibody-Dependent Cell-Mediated Cytotoxicity (ADCC) and Cytokine Secretion by Resting and Cytokine-Preactivated Natural Killer (NK) Cells
Source: PLoS One. 2015 Apr 27;10(4):e0124677. doi: 10.1371/journal.pone.0124677 (PMC4411161; doi:10.1371/journal.pone.0124677)
Supplement: S1 Fig — Both 293FT cells and 293FT-M2 cells exhibited very similar side scatter and forward scatter, indicating that the expression of M2 did not significantly changed the size and complexity of 293FT cells. Similarly, overnight influenza infection did not significantly change the size and complexity of A549 cells. (DOCX) [file pone.0124677.s001.docx]

**Figure S1:**

**0**

**50K**

**100K**

**150K**

**200K**

**250K**

**0**

**50K**

**100K**

**150K**

**200K**

**250K**


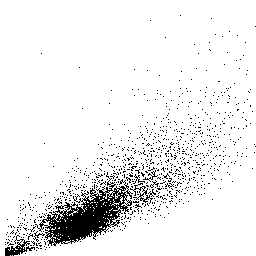


**0**

**50K**

**100K**

**150K**

**200K**

**250K**

**0**

**50K**

**100K**

**150K**

**200K**

**250K**


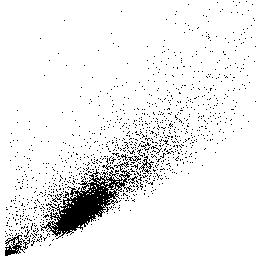


**293FT**

**293FT-M2**

**Forward**

**Scatter**

**S**

**i**

**d**

**e**

**S**

**c**

**a**

**t**

**t**

**e**

**r**

**Figure S1.** **Dot plots of forward and side scatter of the 293FT cells and 293FT-M2 cells (upper panels), and uninfected and influenza infected A549 cells (lower panels).** Both 293FT cells and 293FT-M2 cells exhibited very similar side scatter and forward scatter, indicating that the expression of M2 did not significantly changed the size and complexity of 293FT cells. Similarly, overnight influenza infection did not significantly change the size and complexity of A549 cells.
